# Global strategy for women’s health: use of clinical simulation in gestational risk assessment

**DOI:** 10.1590/0034-7167-2025-0333

**Published:** 2026-07-31

**Authors:** Sheyla Costa de Oliveira, Mônica Oliveira Batista Oriá, Kleyde Ventura de Souza

**Affiliations:** I Universidade Federal de Pernambuco. Recife, Pernambuco, Brazil; II Universidade Federal do Ceará. Fortaleza, Ceará, Brazil.; III Universidade Federal de Minas Gerais. Belo Horizonte, Minas Gerais, Brazil

Women’s health is a central priority on global public health agendas. Within public policy frameworks, prenatal care is a well-established strategy aimed at enhancing best practices for a positive pregnancy experience. The Childbirth and Birth Humanization Policy (2000) and the Stork Network Strategy (2011) aimed to ensure humanized, equitable, and high-quality care. Currently, the Alyne Network (2024) integrates a series of actions aimed at reducing maternal mortality, including the organization of prenatal care, the prevention of complications during pregnancy, and risk assessment with classification^(1)^.

With the formulation of the Sustainable Development Goals (SDGs), a global action plan began to include health and well-being, encompassing improvements in maternal health indicators. The World Health Organization (WHO) states that 92% of maternal deaths occur in low-development countries, reflecting social, regional, and racial inequalities. Brazil has committed to reducing the maternal mortality ratio (MMR) to 30 deaths per 100,000 live births (LBs) by 2030. In 2023, the MMR was 50.9/100,000 LBs. This text advocates for the need for the country to advance in the adoption of safe practices, aiming at reducing maternal mortality as a global strategy and reducing inequalities.

In this context, gestational risk assessment is a safe practice and should begin even in the pre-conception period, considering reproductive risk and health conditions. This type of assessment has a direct effect on reducing complications and adverse events. This is a systematic strategy that identifies biological and non-biological health factors, guides risk stratification for individualized care, enables timely referrals according to the healthcare network flow, and should be carried out by healthcare professionals responsible for prenatal consultations. Clinical, obstetric, social, and epidemiological criteria are described in official instruments such as the Pregnancy Booklet and the Primary Care Prenatal Record (e-SUS/PEC).

In the context of global health, what is required is not only the implementation of effective public policies, but also reflection on professional training processes capable of educating healthcare professionals to deal with the complex demands of obstetric care, committing to safe and humanized care. Healthcare and educational institutions have developed integrated educational strategies focused on safe and harm-free care, recognizing the challenge of the Brazilian Unified Health System as the organizer of professional training. In teaching and learning methods, clinical simulation is configured as a resource for pedagogical innovation and an educational strategy.

In obstetric care, healthcare professionals face the challenge of developing competencies for assessing gestational risk using clinical reasoning and judgment in their work and care processes. Among the limitations, skills related to problem-solving and decision-making stand out.

For the development of clinical simulation, it is necessary to incorporate good learning practices, with intentional, systematic, flexible, and cyclical planning. Following these standards helps in the development of relevant and educationally consistent experiences^(2)^. This practice is encouraged by the WHO as one of the global health strategies, aiming to empower professionals in accordance with maternal safety and quality of care.

The development of clinical simulation scenarios should be understood as a pedagogical practice linked to undergraduate, graduate, and healthcare services. The scenarios must be carefully planned and validated, with the definition of central learning objectives and the assessment of learners’ needs, always guided by public health and education guidelines and policies^(3,4)^. In the scenario script for gestational risk assessment, it is recommended to describe a clinical situation within a broader context that considers the social determinants of health. Clinical simulation should enable clinical judgment with gestational risk classification and stratification. Among the scenarios most described in the literature are obstetric emergencies and postpartum hemorrhage.

In light of this, future directions are suggested for professional training and development in a safe manner, encompassing reflections on competencies and skill performance, as well as the development of critical thinking for decision-making in gestational risk assessment. Furthermore, it is essential to identify improvements in prenatal care based on feedback, active learning, diversified approaches, and individualized care. In this scenario, nursing plays a fundamental role in promoting maternal health and safety, acting proactively in care practices and innovative educational strategies for qualified and humanized obstetric care, contributing to the achievement of the SDGs.
