# Supplementary material for: Global strategy for women’s health: use of clinical simulation in gestational risk assessment
Source: Rev Bras Enferm. 2026 Jul 31;79:e20250333. doi: 10.1590/0034-7167-2025-0333 (PMC13435851; doi:10.1590/0034-7167-2025-0333)
Supplement: Translation

# Translation: Estratégia global de saúde da mulher: uso da simulação clínica na avaliação do risco gestacional

**DOI:** 10.1590/0034-7167-2025-0333pt

**Authors:** Sheyla Costa de Oliveira, Mônica Oliveira Batista Oriá, Kleyde Ventura de Souza

**Affiliations:** I Universidade Federal de Pernambuco. Recife, Pernambuco, Brasil; II Universidade Federal do Ceará. Fortaleza, Ceará, Brasil; III Universidade Federal de Minas Gerais. Belo Horizonte, Minas Gerais, Brasil

A saúde das mulheres é uma prioridade central nas agendas globais de saúde pública. Nas estruturas das políticas públicas, a atenção pré-natal é uma estratégia consolidada, com o objetivo de potencializar as boas práticas para uma experiência positiva na gravidez. A Política de Humanização do Parto e Nascimento (2000) e a Estratégia Rede Cegonha (2011) tinham como diretriz assegurar uma assistência humanizada, equânime e de qualidade. Atualmente, a Rede Alyne (2024) integra uma série de ações voltadas à redução da morte materna, incluindo a organização do pré-natal, a prevenção das intercorrências na gestação e a avaliação com classificação de risco^(1)^.

Com a formulação dos Objetivos de Desenvolvimento Sustentável (ODS), um plano de ação global passou a inclui a saúde e bem-estar, contemplando melhorias nos indicadores de saúde materna. A Organização Mundial da Saúde (OMS) afirma que 92% das mortes maternas ocorrem em países com baixo desenvolvimento, refletindo desigualdades sociais, regionais e raciais. O Brasil assumiu o compromisso de reduzir a razão de mortalidade materna (RMM) para 30 óbitos por 100 mil nascidos vivos (NV s) até 2030. Em 2023, a RMM foi de 50,9/100.000 NVs. Este texto advoga a necessidade de o país avançar na adoção de práticas seguras, visando à redução da mortalidade materna como estratégia global e redução de desigualdades.

Nesse contexto, a avaliação do risco gestacional é uma pratica segura e deve iniciar ainda no período pré-concepcional, considerando o risco reprodutivo e condições de saúde. Esse tipo de avaliação tem efeito direto na redução de agravos e eventos adversos. Trata-se de estratégia sistemática que identifica fatores de saúde biológicos e não biológicos, orienta a estratificação de risco para um cuidado individualizado, possibilita encaminhamentos oportunos conforme o fluxo da rede de assistência e deve ser realizada por profissionais de saúde responsáveis pelas consultas de pré-natal. Os critérios clínicos, obstétricos, sociais e epidemiológicos estão descritos em instrumento oficiais como a Caderneta da Gestante e a Ficha de Pré-natal da Atenção Primária (e-SUS/PEC).

No contexto da saúde global, exigem-se não apenas a implementação de políticas públicas efetivas, mas também reflexões sobre os processos de formação profissional, capazes de educar profissionais de saúde para lidar com as demandas complexas da assistência obstétrica, comprometendo-se com o cuidado seguro e humanizado. Instituições de saúde e de ensino têm desenvolvido estratégias educacionais com ações integradas, voltadas à assistência segura e livre de danos, reconhecendo o desafio do Sistema Único de Saúde como ordenador da formação profissional. Nos modos de ensinar e aprender, a simulação clínica se configura como recurso de inovação pedagógica e estratégia educacional.

Na assistência obstétrica, os profissionais de saúde enfrentam o desafio de desenvolver competências para avaliação do risco gestacional utilizando raciocínio e julgamento clínico em seu processo de trabalho e cuidado. Entre as limitações, destacam-se as habilidades relacionadas à resolução de problemas e à tomada de decisão.

Para o desenvolvimento da simulação clínica, é necessário incorporar boas práticas de aprendizagem, com planejamento intencional, sistemático, flexível e cíclico. O seguimento desses padrões auxilia no desenvolvimento de experiências relevantes e educacionalmente consistentes^(2)^. Tal práxis é estimulada pela OMS como uma das estratégias globais de saúde, visando capacitar profissionais em conformidade com a segurança materna e a qualidade do cuidado.

A elaboração de cenários de simulação clínica deve ser entendida como uma prática pedagógica articulada à graduação, à pós-graduação e aos serviços de saúde. Os cenários devem ser cuidadosamente planejados e validados, com a definição de objetivos centrais de aprendizagem e a avaliação das necessidades dos educandos, sempre orientados pelas diretrizes e políticas públicas de saúde e educação^(3,4)^. No *script* de cenário para avaliação do risco gestacional, recomenda-se descrever uma situação clínica em um contexto ampliado que considere os determinantes sociais da saúde. A simulação clinica deve possibilitar o julgamento clínico com classificação e estratificação do risco gestacional. Entre os cenários mais descritos na literatura, estão as emergências obstétricas e hemorragias pós-parto.

Diante disso, sugerem-se direções futuras para a formação e capacitação profissional de forma segura, contemplando reflexões sobre competências e desempenho de habilidades, bem como o desenvolvimento do pensamento crítico para tomada de decisão na avaliação do risco gestacional. Além disso, é essencial identificar melhorias da assistência pré-natal com base em *feedback*, aprendizagem ativa, abordagens diversificadas e singularização do cuidado. Nesse cenário, a enfermagem exerce papel fundamental na promoção da saúde e da segurança materna, atuando com protagonismo nas práticas de cuidado e em estratégias educativas inovadoras para uma assistência obstétrica qualificada e humanizada, contribuindo para o alcance dos ODS.

# Translation: Estrategia global para la salud de la mujer: uso de la simulación clínica en la evaluación del riesgo gestacional

**DOI:** 10.1590/0034-7167-2025-0333es

**Authors:** Sheyla Costa de Oliveira, Mônica Oliveira Batista Oriá, Kleyde Ventura de Souza

**Affiliations:** I Universidade Federal de Pernambuco. Recife, Pernambuco, Brasil; II Universidade Federal do Ceará. Fortaleza, Ceará, Brasil. ^III^ Universidade Federal de Minas Gerais. Belo Horizonte, Minas Gerais, Brasil

La salud de la mujer es una prioridad central en las agendas globales de salud pública. Dentro de las políticas públicas, la atención prenatal es una estrategia consolidada que busca mejorar las mejores prácticas para una experiencia de embarazo positiva. La Política de Humanización del Parto y el Nacimiento (2000) y la Estrategia de la Red Cigüeña (2011) buscaban garantizar una atención humanizada, equitativa y de alta calidad. Actualmente, la Red Alyne (2024) integra una serie de acciones para reducir la mortalidad materna, incluyendo la organización de la atención prenatal, la prevención de complicaciones durante el embarazo y la evaluación de riesgos con clasificación^(1)^.

Con la formulación de los Objetivos de Desarrollo Sostenible (ODS), se inició un plan de acción global que incluyó la salud y el bienestar, incluyendo mejoras en los indicadores de salud materna. La Organización Mundial de la Salud (OMS) afirma que el 92 % de las muertes maternas se producen en países de bajo desarrollo, lo que refleja desigualdades sociales, regionales y raciales. Brasil se ha comprometido a reducir la tasa de mortalidad materna (TMM) a 30 muertes por cada 100.000 nacidos vivos (NVs) para 2030. En 2023, la TMM fue de 50,9/100.000 NVs. Este texto aboga por la necesidad de que el país avance en la adopción de prácticas seguras, con el objetivo de reducir la mortalidad materna como estrategia global y reducir las desigualdades.

En este contexto, la evaluación del riesgo gestacional es una práctica segura y debe comenzar incluso en el período preconcepcional, considerando el riesgo reproductivo y las condiciones de salud. Este tipo de evaluación tiene un efecto directo en la reducción de complicaciones y eventos adversos. Esta es una estrategia sistemática que identifica factores de salud biológicos y no biológicos, orienta la estratificación del riesgo para una atención individualizada, permite derivaciones oportunas según el flujo de la red de atención y debe ser implementada por profesionales de la salud responsables de las consultas prenatales. Los criterios clínicos, obstétricos, sociales y epidemiológicos se describen en instrumentos oficiales como la Cartilla de Embarazo y el Registro Prenatal de Atención Primaria (e-SUS/PEC).

En el contexto de la salud global, lo que se requiere no es sólo la implementación de políticas públicas efectivas, sino también la reflexión sobre procesos de formación profesional capaces de formar a los profesionales de la salud para enfrentar las complejas demandas de la atención obstétrica, comprometiéndose con una atención segura y humanizada. Las instituciones sanitarias y educativas han desarrollado estrategias educativas integradas centradas en una atención segura y sin riesgos, reconociendo el desafío del Sistema Único de Salud como organizador de la formación profesional. En los métodos de enseñanza y aprendizaje, la simulación clínica se configura como un recurso para la innovación pedagógica y una estrategia educativa.

En la atención obstétrica, los profesionales de la salud se enfrentan al reto de desarrollar competencias para evaluar el riesgo gestacional mediante el razonamiento y el juicio clínicos en sus procesos laborales y asistenciales. Entre las limitaciones, destacan las habilidades relacionadas con la resolución de problemas y la toma de decisiones.

Para el desarrollo de la simulación clínica, es necesario incorporar buenas prácticas de aprendizaje, con una planificación intencional, sistemática, flexible y cíclica. Seguir estos estándares facilita el desarrollo de experiencias relevantes y con coherencia educativa^(2)^. Esta práctica es incentivada por la OMS como una de las estrategias de salud global, con el objetivo de empoderar a los profesionales en materia de seguridad materna y calidad de la atención.

El desarrollo de escenarios de simulación clínica debe entenderse como una práctica pedagógica vinculada a los servicios de pregrado, posgrado y salud. Los escenarios deben planificarse y validarse cuidadosamente, definiendo los objetivos centrales de aprendizaje y evaluando las necesidades de los estudiantes, siempre con base en las directrices y políticas de salud pública y educación^(3,4)^. En el guion de escenarios para la evaluación del riesgo gestacional, se recomienda describir una situación clínica dentro de un contexto más amplio que considere los determinantes sociales de la salud. La simulación clínica debe facilitar el juicio clínico con la clasificación y estratificación del riesgo gestacional. Entre los escenarios más descritos en la literatura se encuentran las emergencias obstétricas y la hemorragia posparto.

En vista de esto, se sugieren futuras orientaciones para la formación y el desarrollo profesional de forma segura, que abarquen la reflexión sobre las competencias y el desempeño de habilidades, así como el desarrollo del pensamiento crítico para la toma de decisiones en la evaluación del riesgo gestacional. Asimismo, es fundamental identificar mejoras en la atención prenatal basadas en la retroalimentación, el aprendizaje activo, los enfoques diversificados y la atención individualizada. En este contexto, la enfermería desempeña un papel fundamental en la promoción de la salud y la seguridad maternas, actuando proactivamente en las prácticas de atención y en estrategias educativas innovadoras para una atención obstétrica cualificada y humanizada, contribuyendo así al logro de los ODS.
